# The use of evidence in public governmental reports on health policy: an analysis of 17 Norwegian official reports (NOU)

**DOI:** 10.1186/1472-6963-9-177

**Published:** 2009-09-28

**Authors:** Simon Innvær

**Affiliations:** 1 Faculty of Social Sciences, Oslo University College, PO Box 4, St Olavs plass, 0130 Oslo, Norway

## Abstract

**Background:**

Governments increasingly require policy documents to be evidence-based. This paper analyses the use of scientific evidence in such documents by reviewing reports from government-appointed committees in Norway to assess the committees' handling of questions of effect.

**Methods:**

This study uses the 'Index of Scientific Quality' (ISQ) to analyse all Norwegian official reports (NOUs) that were: (1) published by the Norwegian Ministry of Health and Care Services during 1994-1998 (N = 20); and (2) concerned with questions of effect either because these were included in the mandate or as a result of the committee's interpretation of the mandate. The ISQ is based on scientific criteria common in all research concerning questions of effect. The primary outcome measure is an ISQ score on a five-point scale.

**Results:**

Three reports were excluded because their mandates, or the committees' interpretations of them, did not address questions of effect. For the remaining 17 NOUs in our study, overall ISQ scores were low for systematic literature search and for explicit validation of research. Two reports had an average score of three or higher, while scores for five other reports were not far behind. How committees assessed the relevant factors was often unclear.

**Conclusion:**

The reports' evaluations of health evidence in relation to questions of effect lacked transparency and, overall, showed little use of systematic processes. A systematic, explicit and transparent approach, following the standards laid down in the ISQ, may help generate the evidence-based decision-making that Norway, the UK, the EU and the WHO desire and seek. However, policy-makers may find the ISQ criteria for assessing the scientific quality of a report too narrow to adequately inform policy-making.

## Background

Nearly 30 years ago, Lindblom and Cohen stated: '... in public policy making, many suppliers and users of social research are dissatisfied, the former because they are not listened to, the latter because they do not hear much they want to listen to' [1]. Twenty years later, the British government asked for policy that was 'shaped by the evidence rather than a response to short-term pressures' [2]. This quest for evidence by the British government exemplified a new trend in modern policy-making and, since the early 1990s, the success of evidence-based medicine has generated calls for, and discussions of, evidence-based policy-making [3-11]. This trend has been encouraged by international institutions such as the EU and WHO [12-14].

A call for evidence-based policy-making is also a call for the use of scientific methods in data collection and in the validation of information. Whenever policies are discussed, the question is often simply, "What will work?" Accordingly, when making priorities and formulating healthcare policies, a key factor will be evidence of their effects. A transparent and explicit approach is essential for reliable, valid and rational decision-making.

In interview studies, policy-makers state that three factors will facilitate their use of scientific evidence in policy decisions: high quality research; the inclusion of data on effectiveness; and summaries with clear recommendations [1,15-18]. If research studies are to have a greater impact in decision-making, this means it is important to assess the quality of such a vital element in official policy documents.

Previous studies of health policy documents commissioned in the UK and elsewhere and by the WHO have investigated the consistency of suggested policies with evidence gathered in the field [14,19-23]. Studies of documents commissioned in the UK have revealed a lack of consistency between recommendations and best evidence [20].

In 2002, the Norwegian government stated that it wished to adopt the evidence-based approach to policy-making[12]. In Norway, the government and the parliament frequently commission comprehensive reports, known as Norwegian official reports (NOUs), which deal with complex policy questions of national importance [24]. In such cases the government nominates a committee to write the report on the basis of a set of relevant questions (the mandate). NOUs are clearly the government policy documents that make most reference to research evidence. Similar reports exist in Sweden and in both countries these reports resemble a hybrid of British green papers and commissioned reports. There are many studies of health policy-makers' perceptions of their use of evidence, but only few studies of their actual use of evidence [15]. This paper presents an empirical study that examines how scientific evidence is used in policy documents by reviewing reports from government-appointed committees in Norway to assess their handling of questions of effect.

## Methods

All NOUs published by the Norwegian Department of Health and Care Services during 1994-1998 were collected and those that reported evidence of health effects were included in this study. Of the 20 NOUs published during the study period, three were excluded either because the mandates did not address questions of effect or because the committees did not interpret the mandates as doing so, resulting in a total of 17 analysed NOUs. A full list of titles appears in Table 1.

**Table 1:** NOUs from the Department of Health and Care Services, from 1994 through 1998

1. From paid work to retirement. **NOU 1994: 2**	2. The use of cells and tissues from aborted embryos. **NOU 1994: 22**
3. Programme for health and social services for the Sami population in Norway. **NOU 1995: 6**	4. Regional institutions for patients in need of long-term care. **NOU 1995: 14**
5. Electromagnetic fields and public health. **NOU 1995: 20**	6. Alcohol policy -- in need of change? **NOU 1995: 24**
7. The patient first: leadership and management of hospitals. **NOU 1997: 2**	8. Framework for sale and distribution of pharmaceuticals. **NOU 1997: 6**
9. Alternative medicine. **NOU 1998: 21**	10. Pharmaceuticals: priorities and policies. **NOU 1997: 7**
11. Priorities revisited -- guidelines for making priorities in Norwegian healthcare. **NOU 1997: 18**	12. Care and knowledge: the Norwegian cancer programme. **NOU 1997: 20**
13. Eradication of tuberculosis? A strategy for future tuberculosis control. **NOU 1998: 3**	14. Air ambulances in Norway. **NOU 1998: 8**
15. Emergency needs. Professional skills for emergency preparedness. **NOU 1998: 9**	16. The Alta battalion. **NOU 1998: 12**
17. Everyone is needed. **NOU 1998: 18**	

Excluded NOUs (because mandates did not address questions of effect):

Co-ordination of public pensions and insurance payments. **NOU 1995: 29**

Financing and out-of-pocket payment for care services. **NOU 1997: 17**

Access to health registers. **NOU 1997: 26**

### Analytical strategy

Each NOU included within the study was circulated between four researchers (see the acknowledgements at the end of the paper) until general agreement was reached regarding the relevant questions of effect in the mandate or the committee's interpretation of the mandate. The same researchers then worked in pairs to score all the reports using a predefined set of evaluation questions. Group discussions were then held to resolve opposing views. We also analysed the composition of each committee, i.e., the relative importance of the roles of researchers, policy-makers and representatives from interest organisations.

The predefined questionnaire for evaluating the NOUs consisted of four main components (table 2).

**Table 2:** The four main components For evaluating reports

**1. The mandate's description of the task**	
Was the committee asked to evaluate: (Yes/No)	
A. The extent and the seriousness of the problem?	B. The effectiveness of services in meeting needs created by the problem?
C. Alternative services?	D. Economic consequences?
E. Values, such as the preferences of patients or ethical considerations?	
**2. The strategy used by the committee to gather research information **(Yes/No)	
A. Did the report state that it was based upon research?	B. Did the report state how research was identified?
**3. The use of evidence in recommendations and in the summary **(Score 1-5)	
A. Recommendations: Does the committee clearly state how it weighed up health needs, the effectiveness of treatment, economic concerns, and other values?	B. Is the summary clearly structured and easily understood by non-professionals?
**4**. **Evaluation of the quality of scientific evidence **(Score 1-5)	
A. Relevance: Does the report make it clear for whom the results are relevant?	B. Documentation: Does the presented evidence rely on research, and are references given?
C. Validity: Is the assessment of the validity of the evidence clear and well-founded?	D. Size of effects: Is the size of effects clearly described?
E. Precision: Are confidence intervals identified and evaluated when relevant?	F. Consistency: Are the findings consistent?

G. Consequences: Are the main consequences identified and assessed?	H. Overall quality: What is the overall scientific quality?

The first component, concerning the mandate's description of the task, was included to clarify whether the committee was supposed to evaluate evidence of effect. The purpose of the second component, concerning the strategy used by the committee to gather research information, was to ascertain whether the intentions of the committee were research-based, how the committee proceeded to gather information, and the sources of its data.

The third component concerned the use of evidence in the report's recommendations and summary. Research suggests that policy-makers often rely heavily on these aspects of reports, making an assessment of their scientific quality particularly important [15]. The special guidelines used to address this third component of the evaluation appear in Table 3.

**Table 3:** Questions concerning recommendations and summaries

*Question A* In making its recommendations, did the committee clearly state how it weighed up health needs, effectiveness, resources and values?				
No	Partial		Yes	
The committee does not state how it weighed up health needs, effectiveness, resources other values.	The committee made some explicit statements, but these are not sufficient for a full understanding of how the committee reached its conclusions.		There was an explicit evaluation of health needs, effectiveness, resources and values	
1	2	3	4	5
**Question B** Is the summary clearly structured, and can non-professionals easily understand it?				
No	Partial		Yes	
The content of the report is not clearly described. The summary is difficult to read, unstructured, or does not provide a good account of the content (too long, too short).	The content of the report is, to some extent, presented in the summary, but there are some shortcomings in clarity (difficult language) and structure. The summary is either too long or too short.		The summary is easy to read and and well structured. The summary is neither too long nor too short.	

1	2	3	4	5

The fourth component concerned the quality of the scientific evidence. To assess the use of scientific evidence in the reports, we applied the Index of Scientific Quality (ISQ) [25,26]. The ISQ is based on common scientific criteria in all research based on rigorous methods that aims to answer questions of effect. The ISQ criteria are presented in Table 4.

**Table 4:** Questions Concerning evaluation of the quality of scientific evidence

**Question A. Relevance: Is it clear to whom the information in the report applies?**				
No	Partial		Yes	
Potentially misleading	Minor lack of clarity.		Minimal ambiguity	
1	2	3	4	5
***Question B. Documentation: Does the presented evidence rely on research, and are references given?***				
No	Partial		Yes	
Potentially misleading	Statements are attributed to sources, but the underlying evidence is ambiguous		The evidence underlying the main points is clearly cited	
1	2	3	4	5
***Question C. Validity: Is the assessment of the credibility of the evidence clear and well-founded?***				
No	Partial		Yes	
Not done or potentially misleading	Study design or type of evidence reported but not properly assessed		Strengths of the research methods adequately assessed	
1	2	3	4	5
***Question D. Size of effect: Is the strength of the findings (effects) clearly reported?***				
No	Partial		Yes	
Not done or potentially misleading	The magnitude of effects is reported incompletely or ambiguously		Magnitude of effects clearly reported	
1	2	3	4	5
***Question E. Precision. Are confidence intervals identified and evaluated when relevant?***				
No	Partial		Yes	
Not done or potentially misleading	Indirectly or incompletely		Confidence intervals adequately assessed	
1	2	3	4	5
***Question F. Consistency: Is the consistency of the evidence (between studies) considered?***				
No	Partial		Yes	
Not done or potentially misleading	More than one study is discussed, but consistency is not clearly reported		Number of studies and consistency clearly reported	
1	2	3	4	5
***Question G. Consequences: Are the main consequences (benefits, risks and costs) identified and assessed****?*				
No	Partial		Yes	
Potentially misleading	Important consequences are not considered		Most important consequences are are clearly identified	
1	2	3	4	5
***Question H. Overall quality: Based on the answers to the above questions, how would you rate the overall scientific quality?***				
Low	Moderate		High	
Critical or extensive shortcomings	Potentially important but not critical shortcomings		Minimal shortcomings	

1	2	3	4	5

The index is based on a five-point scale, with a score of 5 representing the highest level of scientific quality. According to the ISQ criteria for documentation (question B), clear references to the evidence will score 4 or 5, while partly unclear or definitely unclear references to evidence will score 2 or 3, depending upon the degree of uncertainty. When references to evidence are potentially misleading, the ISQ score will be 1 or 2. Table 4 shows the guidelines used for scoring each of the eight questions concerning the committees' handling of scientific evidence. Reports of low overall quality will score 1 or 2, indicating extensive shortcomings in relation to several ISQ criteria. Reports of high overall quality, with few shortcomings, will score 4 or 5.

### Limitations of this study

Previously the ISQ has mainly been used by studies evaluating health reporting in newspapers. We know of no other studies that have used the ISQ to evaluate governmental reports. A few reservations are relevant regarding the use of the index in this context. Firstly, public policy formation is clearly much more complicated than the discussion here might suggest. In addition to scientific evidence, policy-makers need to weigh up different factors such as the values of stakeholders, budgets, cost-effectiveness, broader perspectives, political decisions and aims, other, perhaps more relevant, evidence than the existing evidence of effect, prudent practices, and commonsense understandings that are obvious, but not supported by research evidence. As a consequence, evidence of effect will only form one aspect of the decision-making process when evidence-based policies are made [8,27]. Discussions of other approaches to evaluating evidence are interesting, but outside the scope of this paper [9,11,28-41].

Secondly, questions may be raised regarding the sensibility of the ISQ. A comprehensive sensibility analysis of the ISQ [25,26] found the index to be acceptably reliable and credible. Only one major problem was identified: the need to apply judgment when awarding scores. We also encountered this problem, but were able to resolve our initial and minor disagreements through discussion.

Thirdly, a more contextual evaluation of the variations in scores, or treating each NOU as a separate case, could give important insights into the nature of the variations. An example of this problem arises in connection with one of the NOUs about the Norwegian cancer programme, where narrow questions of effect can only make a minor contribution to a broad mandate.

Finally, the analysed reports are not the most recent ones, so they may not be representative for recent NOUs. And a move to promote evidence-based policy making in Norway appears to have been made in January 2004, with the formation of an institution called The Norwegian Knowledge Centre for the Health Services (NKCHS). The Centre is organised and funded by the Norwegian Directorate of Health. NKCHS is meant to be scientifically and professionally independent. The Centre has two overarching tasks: The first is to promote evidence-based policy making, by producing health technology assessment reports, systematic reviews, overviews and reports that provide policy makers with early warnings. The second task is to support health services at all levels to incorporate evidence into their practices.

The NOUs published during 1994-1998 seem to be representative of the 19 NOUs published from 1999 to the present. There have been no significant changes in the formal framework for preparing these reports, although some minor revisions were implemented in 2005. Only a shallow investigation of the 19 NOUs published since 1999 is necessary to confirm that when committees interpret their mandates and design their reports, they do not utilize a systematic approach that is similar to the ISQ or familiar evidence-based approaches. Generally speaking it is still the government-appointed committee that determines the methods and procedures used to produce the report. Only one of the NOUs published after 1998 reported that the committee preparing the report asked the NKCHS for a review of the relevant research. The NKCHS responded by stating that such a report would take the Centre from 6 to 24 months to produce. The committee deemed this to be beyond the framework provided to it to complete its work.

## Results

The themes of the reports varied from broad policy issues, such as the setting of healthcare priorities, to narrower issues such as the health consequences of particular stimuli (e.g., electromagnetic fields). Some reports spanned a number of topics, such as those covering health and social services among the Sami population or public health services in local communities.

### Task specification in the mandates

Some mandates specified objectives relating to the organisation of care, e.g., 'How should care be organised to give the best medical and social outcomes for patients with multiple sclerosis?' Other mandates posed more concrete questions: 'Will dramatic experiences during war cause long-term psychiatric problems in soldiers?' 'What is the effect of directly observed treatment for patients suffering from tuberculosis?' The mandates were short, generally a half to one page in length. Most mandates required the committee to consider the following issues: need (15); the effectiveness of relevant interventions (16); and economic implications (15). None of the mandates specified the type or quality of data required. Six of the 17 mandates analysed required a discussion of relevant values, such as the preferences of patients, public considerations, or juridical or ethical consequences.

### Strategies used by committees to gather research information

Some committees consisted mainly of experts in the relevant field, while other committees consulted external experts and included their recommendations as appendices to their reports. Expert participation on the committees did not appear to influence the strategy used for information gathering. While all the reports but one referred to reliance upon research, none of the reports described the procedures or literature searches used to gather research information on the harm or efficacy of relevant interventions. Although one report (on electromagnetic fields) stated that it included all the relevant literature, it provided no information about how the literature search had been conducted. Another report (on complementary medicine) stated that various organisations had been asked to provide relevant publications, but did not state the type of literature requested.

### The use of evidence in recommendations and in the summary

Component 3 in table 2 assesses how the committee evaluated the various considerations of health needs, effectiveness, resources and values when making its final recommendations. The average score was 2.9, indicating medium quality. Two reports scored 3.0 or above. The committees responsible for the 15 remaining reports (88 per cent) gave partly unclear descriptions of how they reached their conclusions. In general the committees provided relatively clear and structured summaries, with an average score of 3.4. Ten NOUs scored 4, while only three scored below 3.0.

### Evaluation of the quality of scientific evidence

**Figure 1:**
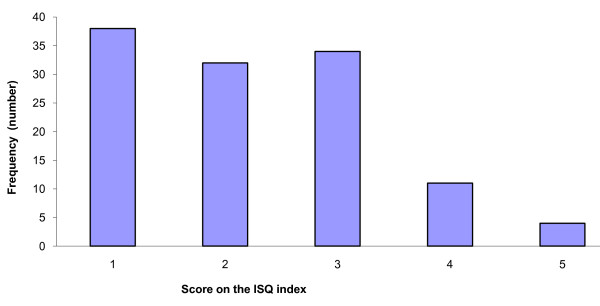
**Scores for all the 119 judgements on scientific quality (1-5 scale) (See box 3 for the 7 judgements for 17 NOUs, N = 119, overall quality not included)**.

The 17 NOUs were evaluated through the application of seven criteria to assess the quality of scientific evidence. This produced 119 evaluations distributed on a five-point scale. The scores for all 119 evaluations appear in Figure 1.

In general, evidence of harm and evidence of effectiveness were presented in combination with information about preferences and problems concerning access to, and availability of, health services. Fifty of the 119 evaluations (42 per cent) resulted in scores ranging between 3 and 5. Only 15 evaluations (13 per cent) resulted in scores exceeding 4.0. Sixty-nine of the 119 evaluations (58 per cent) only scored 1 to 2. In short, these results suggest that more than half of the evaluations identified insufficient descriptions and assessments of the quality of evidence of effect.

**Figure 2:**
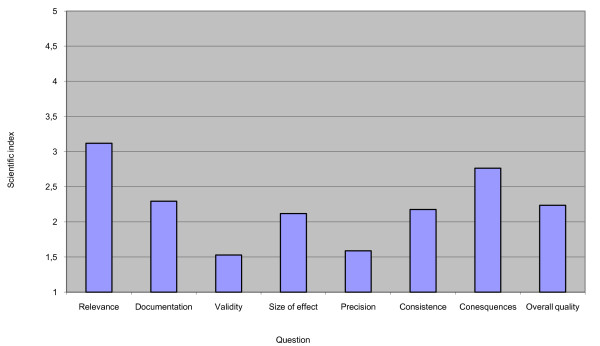
**This figure displays a graphs expressing Average scientific index for the 8 questions on quality**.

Figure 2 shows the average score in relation to each question in the ISQ (see table 4).

Question A asked whether the report made clear *the specific population for whom the report was relevant*. Question A produced an average score of 3.1, the only average score to exceed 3.0. While most reports were partly unclear about the population for whom they were relevant, many also provided important and relevant information. Question B asked *whether the presented evidence relied on research and whether the reports referred properly to the research*. The average score here was 2.3. Only two reports specified references for the most important information.

Question C concerned *the assessment of study quality*. Only two reports provided details of study design and only one described how the committee had assessed the quality of the evidence. The average score here was 1.5. Question D concerned the reporting of the size of the effect and produced an average score of 2.1. Question E asked about *the precision with which the effect had been measured *and produced an average score of 1.6. Many reports described interventions as 'effective', but none reported the magnitude of the most significant effects. Questions C and E produced the lowest scores, both with an average slightly exceeding 1.5.

Only one report attained a score of 5 for its consideration of how the evidence presented related to other findings in the field (question F on attention given to *consistency *between studies). The average score for question F was 2.2. We identified discussions concerning more than one study in five reports. These were awarded scores of 3. The other 11 reports scored only 1 or 2 as they provided either potentially misleading information or no information at all about consistency between studies. As a general comment, committees that, for example, only identify a single research study relevant to their report should at least discuss whether this is the result of a low-quality literature search. In cases where there is only one study dealing with a given question of effect, an evidence-based evaluation should include an assessment of the robustness of the evidence.

Question G dealt with the reports' assessments of the interventions' most significant *consequences related to benefits, risks and costs*. Only two reports scored above 3. The average score was 2.8. Question H on *overall quality *required a global evaluation of each report, resulting in an average score of 2.3.

**Figure 3:**
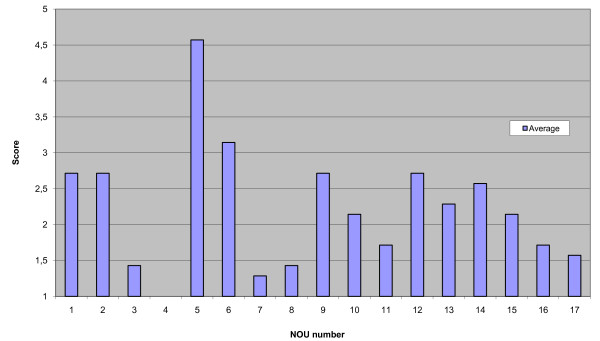
**This figure displays a graph illustrating Average scientific quality for each report**.

Figure 3 shows the average ISQ score for each report.

ISQ scores were generally low. Average scores for seven reports (41 per cent) were above or just under 3.0. These reports generally provided important scientific information regarding questions of effect. Only one report had an average score exceeding 4.0, while one other report scored above 3.0. There was a wide range of average scores, since while one report failed to describe the effectiveness of the services suggested and therefore scored 1 on all questions, another received high scores all round.

## Discussion

Systematic reviews of the outcomes of randomised controlled trials have probably been the key element in the development of evidence-based medicine. Systematic reviews assess search strategies and carry out critical appraisals. The ISQ is based upon the same principles as those underlying systematic reviews. Ministries, the government, and parliament rely on NOUs to provide the most comprehensive advice on subjects of national importance. Our analysis suggests that more than half of NOUs dealing with healthcare contain inadequate descriptions and assessments of the quality of the evidence of effect. We are not suggesting that NOUs provide the public with low-quality reports, merely that their scientific quality regarding questions of effect is low.

Our study suggests that committees prioritise policy relevance, as the NOUs included in the study primarily focused on policy relevance and policy implications. In both respects we found the reports' use of scientific evidence to be generally mediocre. We identified a lack of clarity regarding both the reports' primary intended audience and the identification and assessment of the main consequences of the study. There was insufficient treatment of validity and precision in relation to hard science questions. This may arise from a belief among the authors of reports that policy-makers are uninterested in the science underlying committee recommendations. Committees may also not consider their time and resources best spent teaching policy-makers about scientific standards.

It is impossible to be sure why committees give lower priority to hard science questions, since we have no data from interviews with committee members. We do know, however, from a systematic review of interview studies concerning health policy-makers' perceptions of their use of evidence, that policy-makers want summaries that make clear recommendations [15]. The committees may focus on relevance and consequences simply because they believe this is what policy-makers primarily want and find useful in decision-making. There is some support for this inference in our finding that the scientific quality of the reports is highest where evidence is used in the recommendations and in the summary. Our findings suggest that both policy-makers and experts tend to prioritise summaries and recommendations, rather than the hard science underlying them, since experts' participation on committees did not seem to influence the strategy used to gather research information.

The fact that seven reports obtained an average ISQ score of below 2.0 suggests that committees give insufficient attention to the assessment of scientific evidence. In other words, policy-makers must look elsewhere for transparent descriptions of search strategies for literature, references to evidence gathered in the field, and assessments of the evidence of effect. Our raw data indicates that questions of effect escaped the committees' attention in three of the seven reports, making their ISQ scores of 1 or 2 hardly surprising. We have no information about why some of the committees largely avoided questions of effect.

The mandates typically posed broad questions, rather than clear-cut questions of effect. Although NOUs were only included in our study if questions of effect were relevant to their mandates, these questions of effect were sometimes rather implicit and were always connected to broader issues and to a broader mandate. Since NOUs are intended to deal with questions of national importance, their mandates will naturally be broad. It is easy to imagine the broad mandate for the NOU report 'Care and knowledge: the Norwegian Cancer Programme', which deals with a variety of topics, such as: definitions of cancer; cancer as a national problem; the social consequences of cancer; care structures and processes for cancer sufferers; and the administrative and organisational consequences. Within the time span allotted for producing a report of this nature, it is very difficult for a committee to carry out a comprehensive review of questions of effect.

When reviewing cancer treatments, it is also of course difficult to decide where to focus. Even if there is a decision to focus on breast cancer (instead of, for example, lung cancer or childhood cancer), it will still be necessary to select from the many different questions of effect in the treatment of breast cancer. Even a narrow focus on 10 different questions of effect with a protocol in the Cochrane library will still exclude more than 50 other protocol questions in the same database [42]. Let us suppose that an NOU dealing with a Norwegian cancer programme dealt with 10 questions of effect in the treatment of breast cancer. Even if the report were written in such a way as to receive top ISQ scores, it would still tell us little about the Norwegian cancer programme. An NOU that answers a broad mandate by focusing narrowly on questions of effect is less useful for policy-makers.

## Conclusion

Politicians, scientists, bureaucrats, experts and lay people often have (quite legitimately) differing interests. If public governmental reports refer to evidence in an explicit and transparent way, readers will largely be able to make reliable judgments by comparing the pros and cons apparent from the evidence. Explicit and transparent references to supporting evidence will assist in the identification of bias rooted in special interests in the recommendations of governmental reports.

This study shows that NOUs, in relation to their evaluation of health effects, lack transparency and, overall, show few signs of the use of systematic processes as far as scientific quality is concerned. A systematic, explicit and transparent approach - in fact, an approach that would generate a high ISQ score - may encourage the development of the evidence-based decision-making that Norway and other nations and international institutions desire and seek.

Some people might object that applying the ISQ criteria would overly narrow the focus of reports and cause the omission of other kinds of information more relevant for policy-makers: instead of promoting democratic debate, application of the ISQ criteria might lead to meritocracy. However, such objections would be convincing only if reports scoring highly on the ISQ criteria were inherently difficult for politicians, bureaucrats, experts and lay people to understand. Instead of rejecting out of hand the possibility of writing understandable reports of high scientific quality, committees should focus on writing reports that are accessible to academics and non-academics alike. Committees that include experts as well as non-experts should make particular efforts in this regard. One possible solution would be to prepare separate reports for different audiences. The process of doing so might help clarify the role of scientists in such committees and protect researchers from having their results abused [29,43].

Research is a cumulative enterprise. Whether the ISQ is useful for policy-making, and whether a narrow focus on questions of effect helps policy-makers make wise decisions remains to be seen. The ISQ cannot and should not exclude politics from evidence-based policy-making. But if calls by governments for evidence-based policy-making are to be taken seriously, we need to develop and test evidence-based approaches such as those endorsed by the ISQ so that evidence-based policy may progress from aspiration to reality.

## Abbreviations

EU: European Union; ISQ: Index of Scientific Quality; NKCHS: The Norwegian Knowledge Centre for the Health Services; NOU: Norwegian Official Reports (Norges Offentlige Utredninger); WHO: World Health Organisation.

## Competing interests

The author declares that he has no competing interests.

## Pre-publication history

The pre-publication history for this paper can be accessed here:


